# Factors associated with sleep quality among Afghan healthcare workers

**DOI:** 10.1002/hsr2.70018

**Published:** 2024-08-29

**Authors:** Ahmad Shoaib Haidarzada, Ahmad Neyazi, Bijaya K. Padhi, Nosaibah Razaqi, Habibah Afzali, Raz Mohammad Tabib, Mahsa Ahmadi, Mehrab Neyazi, Mark D. Griffiths

**Affiliations:** ^1^ Department of Mental Health Herat Regional Hospital Herat Afghanistan; ^2^ Afghanistan Center for Epidemiological Studies Herat Afghanistan; ^3^ Faculty of Medicine Ghalib University Herat Afghanistan; ^4^ Department of Community Medicine and School of Public Health Postgraduate Institute of Medical Education and Research Chandigarh India; ^5^ Scientific Research Committee Afghanistan Medical Students Association Herat Afghanistan; ^6^ Department of Psychology Nottingham Trent University Nottingham UK

**Keywords:** Afghanistan, healthcare workers, sleep quality, Taliban

## Abstract

**Background and Aims:**

Sleep is a complex physiological process during which the body and mind enter a period of rest. For a healthy lifestyle, different cohort groups can be affected in different ways. One such cohort is healthcare workers (HCWs)—an unexplored group in Afghanistan. Therefore, the present study examined the association between a range of sociodemographic factors including having night shift work and sleep quality among Afghan HCWs.

**Methods:**

A cross‐sectional survey was administered in January 2023 among HCWs (*N* = 342) in the Herat province of Afghanistan. The survey examined sleep quality and its association with a range of sociodemographic factors among HCWs. Logistic regression models were used to examine the association of sleep quality with sociodemographic characteristics among HCWs. The Pittsburgh Sleep Quality Index (PSQI) was used to assess the sleep quality among HCWs.

**Results:**

A total of 342 HCWs participated in the present study with an age range of 18 to 60 years. The mean age of the participants was 28.90 years (SD ± 8.10). Just over half of the participants were male (50.3%). Of the 342 participants, 58.5% reported poor sleep quality. Being married status, having higher number of children, not being a doctor, having low economic status, working night shifts, and having a traumatic event occur during past month were among the main variables associated with sleep quality among Afghan HCWs. Multiple regression analysis indicated that having high income [negatively] (AOR = 4.132, *p* = 0.002), working night shifts [positively] (AOR = 0.288, *p* < 0.001), and having a traumatic event occur during past month [positively] (AOR = 0.504, *p* = 0.007) were significantly associated with sleep quality.

**Conclusion:**

The study suggests the need for Afghan health employers to create a healthy work environment that prioritizes the well‐being of their employees by limiting overtime hours, providing rest breaks during long shifts, and ensuring that HCWs have access to necessary resources for coping with work‐related stressors. These policies would promote the overall health and well‐being of the workforce and would likely lead to better patient care outcomes.

## 1 INTRODUCTION

Sleep is defined as a complex physiological process during which the body and mind enter a period of rest.^1^ For a healthy lifestyle, different age groups need different amounts of sleep. More specifically, in a 24‐h period, infants (4–12 months) need 12–16 h of sleep, 1–2‐year‐old children need between 11 and 14 h, 3–5‐year‐old children need 10–13 h, 6–12‐year‐old children need 9–12 h, adolescents (13–18 years) need 8‐10 h, and adults need 7 or more hours.^2^ In addition to sleep quantity, sleep quality is also an important aspect of sleep.^3^ A person's satisfaction with all elements of sleep experience defines their sleep quality.^4^ Sleep quality consists of four characteristics: sleep efficiency, sleep latency (the time it takes one to fall asleep after going to bed), sleep duration, and waking after sleep onset (the amount of time a person spends being awake after falling asleep).^4^ It is important to recognize the signs of poor‐quality sleep which include feeling drowsy despite getting enough sleep, waking up throughout the night and/or exhibiting signs of a sleep disorder such as snoring.^5^ Sleep quality impacts an individual's body, memory, and learning.^6^ A good night's sleep provides favorable impacts such as being well rested and maintaining positive relationships, whereas a poor sleep quality can make individuals irritable, drowsy, and slow in responses.^4^

Sleep quality is associated with work performance such that better sleep at night leads to a more productive work the next day.^7^, ^8^ A study on employees in Saudi Arabia found that a highly significant outcome of poor‐quality sleep was sleeping during work time. Moreover, the study found that those employees who took sleep medicines were more likely to be absent from work or make errors while working.^9^ In another study on the work performance of working mothers in Pakistan, researchers found that sleep deprivation not only negatively impacted work performance but was also associated with workplace deviance.^10^ Studies have shown that individuals with less sleep duration are prone to a faster global cognitive decline^11^ while cognitive abilities are considered predictors of good job performance.^12^ Moreover, workers with insomnia are at a higher risk for injuries in the workplace and this outcome is mediated by workplace cognitive failure.^13^ In relation to shift work, studies have shown that night‐shift workers are considerably more likely to report sleep disturbances, poorer sleep quality, shorter sleep latency, shorter sleep duration, and daytime dysfunction.^14^ Insufficient sleep can lead to complications such as high levels of cortisol which in turn can lead to high blood sugar, high blood pressure, and weight gain, as well as anxiety, depression, and stroke.^15^

Studies have also found an association between poor sleep quality and stressful life events such that the association of stressful events with poor sleep quality is mediated by concentrating on negative content or rumination.^16^ In a study among Chinese adolescents, researchers found that unpleasant life events are associated with sleep quality and that this association was mediated by negative coping styles.^17^ A common symptom of posttraumatic stress disorder (PTSD) is sleep disturbance. Moreover, women are more likely to experience insomnia and repeated nightmares after a traumatic event compared to men.^18^ Research has also shown that individuals with greater childhood trauma can experience poor sleep health in adulthood.^19^ Research conducted on low‐income adults in a rural area of China found that sociodemographic factors such as gender (being female), marital status (being single), and poorer education were associated with sleep quality.^20^ An Iranian study reported that 53.4% of taxi drivers were reported to have a sleep quality disorder.^21^ Research has also found that in later life, poor socioeconomic status can reduce sleep quality and that this association is mediated by depressive symptoms, and that poverty negatively affects sleep duration and the ability to fall asleep.^22^

A meta‐analysis of observational studies has found that insomnia is more prevalent among women compared to men.^23^ Moreover, studies on sleep quality and sociodemographic data have shown that (in general) married individuals experienced greater levels of REM (rapid eye movement) sleep compared to non‐married individuals.^24^ In terms of sleep problems, women experience more sleep problems than men, and the results are consistent for divorced and widowed individuals compared to married ones.^25^ In relation to parenthood, new parents are often extremely sleep‐deprived if their only sleep time option is at night.^26^ After giving birth to a child, both mothers and fathers experience a decline in sleep duration and satisfaction, and this consequence does not recover for up to 6 years.^27^

Other cohorts can also be more susceptible to sleep problems, particularly healthcare workers (HCWs). In a cohort study among HCWs in the United States (US), data reported that 40.9% of hospital workers experienced sleep disorders, and consequently, sleep disorders were found to be associated with adverse safety incidences.^28^ A study conducted in Turkey during the COVID‐19 pandemic showed that those who worked in healthcare facilities had lower sleep quality compared to those working outside the healthcare field.^29^ A review of sleep studies reported that sleep deprivation is common among HCWs and doctors.^30^ Similarly, a meta‐analysis by Salari et al. reported that the prevalence of sleep disturbance among physicians caring for COVID‐19 patients during the pandemic was 41.6%.^31^ In a systematic review, Khaksarian et al. reported a sleep disturbance rate of 58% among medical and allied health professions students in Iran.^32^ A systematic review concerning Chinese healthcare professionals reported that 39.2% had poor quality sleep and that this was worse than in the Chinese general population.^33^ Poor sleep quality was found to be significantly associated with gender (being female), occupation (being a nurse), and working night shifts. Among Chinese HCWs, the prevalence of sleep disturbance among nurses was found to be higher than other HCW cohorts. In a meta‐analysis of observational studies, it was reported that 61% of nursing staff had poor sleep quality.^34^ It was found that being older, having a higher body mass index, and having more work experience were significantly associated with sleep quality.^34^ In a systematic review, it was reported that 43% of nurses had poor sleep quality during the pandemic.^35^ The demanding role of a nurse, marked by irregular shifts and high responsibilities, was identified as a pivotal factor impacting sleep quality, underscoring the critical need for tailored interventions to address the specific sleep‐related concerns of healthcare professionals working during nocturnal hours.^35^

Although there are many studies on the role of sleep and work performance, there are no data available regarding the association of sleep quality and daytime dysfunction among Afghan HCWs. The participants chosen for the present study were HCWs including doctors and other healthcare staff. The study aimed to:

(1) Examine the prevalence of sleep quality among HCWs.
(2) Analyze the association between various sociodemographic factors, including night shift work, and sleep quality among HCWs.

## 2 METHODS

### 2.1 Participants and procedure

A cross‐sectional survey study was conducted among HCWs between January 29, 2022 and February 5, 2023 in the Herat province of Afghanistan. The study's participants were employees of Herat's nine governmental Hospitals serving as the HCWs as well as those working in the management teams. A total of 342 HCWs participated and were recruited using a convenience sampling technique following the distribution of 600 surveys (response rate = 57%). The researchers personally approached the HCWs to participate in this study. The inclusion criteria to participate in the present study were: (i) working in a hospital; (ii) being 18 years old or older; (iii) being able to understand the Dari or Pashto language, and (iv) providing written informed consent to participate in the study.

### 2.2 Measures

The survey used comprised two main sections: the socioeconomic factors section, and the sleep quality assessment section. The socioeconomic section of the survey included the following items: age (in years), gender (male or female), marital status (single or married), number of children (none, 1–5, 6–12 [the use of the second category (1–5 children) aligns with Statista reports, which indicate that the average number of children in an Afghan family is five.^36^ The third category encompasses families with a higher number of children, with participants in the present study reporting a maximum of 12 children]), field of work (doctors or other), economic status (high‐income [more than $300 per month], middle‐income [$100‐$200 per month], low‐income [less than $100 per month]), worked night shifts (yes or no), cigarette smoker (nonsmoker, ex‐smoker or current‐smoker), and traumatic event occurring during the past month (yes or no). The frequency of night shifts for participants in the present study was typically one night‐shift every 3 working days (and each shift lasted the whole 24 h including both day and night).

The sleep quality section included the Pittsburg Sleep Quality Index (PSQI) to assess the sleep quality of participants.^37^ The PSQI comprises 19 items which are self‐rated and six items which are rated by bed‐partner or roommate. However, only the self‐rated items are considered in the scoring process. The 19 items of the PSQI form seven components (subjective sleep quality, sleep latency, sleep duration, habitual sleep efficiency, sleep disturbances, use of sleep medication, and daytime dysfunction), and each item (e.g., *“*Cannot get to sleep within 30 min”) is rated from 0 (“not during the past month”) to 3 (“three or more times a week”). To calculate the global score, the scores of all of the seven components are summed together which results in a range from 0 (“indicating no difficulty”) to 21 (*“*indicating severe difficulty in all components*”).* Participants having a total score of 5 or less were considered as having good sleep quality, and participants with a total score of over 5 were considered to have poor sleep quality. The Cronbach's *α* in the present study was 0.74.

### 2.3 Data analysis

The collected data were entered into the Microsoft Excel 2016. The statistical analysis was carried out using IBM SPSS version 26.0 software. Variables of each part were presented in category form with their frequency in numbers and percentages. Chi‐square tests were performed to evaluate the association between the participants' socioeconomic characteristics and their sleep quality. The significance level was *p *< 0.05.

### 2.4 Ethics

Ethical approval for the study was obtained from the Afghanistan Center for Epidemiological Studies (Ref: #22.006). The participants were briefed on the relevance and objectives of the study. The purpose of the study was explained to each participant. Confidentiality of information was maintained by omitting any personal identifier from the survey. Participants were aware that they could withdraw at any point in time from the study. All methods were carried out in accordance with relevant ethical guidelines and regulations. Informed consent was obtained from all the participants in the present study.

## 3 RESULTS

A total of 342 HCWs participated in the present study with an age range of 18 to 60 years. The mean age of the participants was 28.90 years (SD ± 8.10). Just over half of the participants were male (50.3%). Almost two‐thirds of the participants were married (63.2%). Almost one‐third of the participants were doctors or residents (36.8%). Almost three‐quarters of the participants reported that they worked night‐shifts (74.6%) (Table 1).

**Table 1:** Characteristics distribution of the study sample.

Characteristic	Categories	Number (*N*)	Percentage
Age group	18–29 years	253	74.0
	30–60 years	89	26.0
Gender	Male	172	50.3
	Female	170	49.7
Marital status	Single	126	36.8
	Married	216	63.2
Number of children	None	180	52.6
	1–5 children	149	43.6
	6–12 children	13	3.8
Field of work	Doctors	126	36.8
	Other	216	63.2
Economic status	High income	119	34.8
	Middle income	166	48.5
	Low income	57	16.7
Working night shifts	Yes	255	74.6
	No	87	25.4
Cigarette smoking status	Nonsmoker	312	91.2
	Smoker	30	8.8
Traumatic event occurred during past month	Yes	142	41.5
	No	200	58.5
Total		**342**	**100.0**

More than half of the participants' sleep quality was found to be poor (58.5%). The mean value for subjective sleep quality was 0.96 (SD ± 0.74) which was lower than 50%. The mean value for the habitual sleep efficiency component was found to be the lowest (0.10; SD ± 0.39) (Table 2).

**Table 2:** Prevalence of sleep quality and its components among participants.

Components	Mean/SD
Subjective sleep quality	0.96 ± 0.74
Sleep latency	1.37 ± 0.97
Sleep duration	0.97 ± 0.92
Habitual sleep efficiency	0.10 ± 0.39
Sleep disturbances	1.25 ± 0.70
Use of sleep medication	0.24 ± 0.67
Daytime dysfunction	1.02 ± 0.93
Global score	5.91 ± 3.36
Sleep quality groups	*N* (%)
Good	142 (41.5)
Poor	200 (58.5)

Almost two‐thirds of the married participants were found to have poor sleep quality (62.5%). More than half of participants with middle‐income economic status were found to have poor sleep quality (60.2%) and two‐thirds of participants who were not medical doctors (nurses, midwives, management team) had poor sleep quality (64.4%). There was a significant relationship between sleep quality and (i) marital status (single participants had better sleep quality than the married participants), (ii) number of children (participants with no children had better sleep quality than those with children), (iii) field of work (participants who were doctors had better sleep quality than other HCWs), (iv) economic status (participants with high‐income had better sleep quality), (v) night‐shift work (participants working night‐shifts had poorer sleep quality than those without nightshifts), and (vi) a traumatic event occurring during the past month (participants who experienced a traumatic event during the past month had poorer sleep quality than those who did not) (Table 3).

**Table 3:** Association of sleep quality with participants sociodemographic characteristics.

Characteristic	Categories	Sleep quality		*p* value
		Good	Poor	
		*N* (%)	*N* (%)	
Age group	18–29 years	111 (43.9)	142 (56.1)	0.137
	30–60 years	31 (34.8)	58 (65.2)	
Gender	Male	74 (43.0)	98 (57.0)	0.571
	Female	68 (40.0)	102 (60.0)	
Marital status	Single	61 (48.4)	65 (51.6)	**0.048**
	Married	81 (37.5)	135 (62.5)	
	None	88 (48.9)	92 (51.1)	
Number of children	1–5 children	52 (34.9)	97 (65.1)	**0.006**
	6–12 children	2 (15.4)	11 (84.6)	
Field of work	Doctor	65 (51.6)	61 (48.4)	**0.004**
	Other	77 (35.6)	139 (64.4)	
	High income	67 (56.3)	52 (43.7)	
Economic status	Middle income	66 (39.8)	100 (60.2)	**<0.001**
	Low income	9 (15.8)	48 (84.2)	
Working night shifts	Yes	84 (32.9)	171 (67.1)	**<0.001**
	No	58 (66.7)	29 (33.3)	
Cigarette smoking status	Nonsmoker	130 (41.7)	182 (58.3)	0.860
	Smoker	12 (40.0)	18 (60.0)	
Traumatic event occurred during past month	Yes	42 (29.6)	100 (70.4)	**<0.001**
	No	100 (50.0)	100 (50.0)	
Total		**142** (**41.5)**	**200** (**58.5)**	

Multinomial logistic regression analysis was run to predict good sleep quality comprising the following variables: age group, gender, marital status, number of children, field of work, economic status, working night shifts, cigarette smoking, and traumatic event occurring during the past month. High‐income economic status (adjusted odds ratio [AOR] = 4.132, *p* = 0.002), middle‐income economic status (AOR = 2.385, *p* = 0.039), working night shifts (AOR = 0.288, *p* < 0.001), and a traumatic event occurring during the past month (AOR = 0.504, *p* = 0.007) significantly contributed to the regression model, predicting good sleep quality (Table 4).

**Table 4:** Multinomial logistic regression analysis of sleep quality and participants' characteristics.

Characteristic	AOR [95% CI]	*p* Value
Age	0.978 [0.936, 1.023]	0.339
Gender		
Male	1.586 [0.940, 2.677]	0.084
Female	Reference	
Marital status		
Single	0.896 [0.433, 1.854]	0.768
Married	Reference	
Number of children		
None	1.626 [0.231, 11.453]	0.626
1–5 children	1.204 [0.198, 7.332]	0.841
6–12 children	Reference	
Field of work		
Doctor	1.564 [0.913, 2.676]	0.103
Other	Reference	
Economic status		
High income	4.132 [1.716, 9.945]	**0.002**
Middle income	2.385 [1.043, 5.455]	**0.039**
Low income	Reference	
Working night shifts		
Yes	0.288 [0.165, 0.505]	**<0.001**
No	Reference	
Cigarette smoking		
Nonsmoker	1.789 [0.732, 4.374]	0.202
Smoker	Reference	
Traumatic event occurred during past month		
Yes	0.504 [0.306, 0.828]	**0.007**
No	Reference	

Abbreviation: CI, confidence interval.

## 4 DISCUSSION

The present study identified several sociodemographic factors that were significantly associated with sleep quality among HCWs in Afghanistan. The findings showed that being married, having children, being a HCW other than a doctor, working night shifts, having low economic status, and experiencing a traumatic event in the past month were all significantly associated with sleep quality.

The present study found that married HCWs reported a significantly higher prevalence of poor sleep quality compared to single workers. This contrasts with a study conducted in Bahrain during the COVID‐19 pandemic, which reported that single HCWs had a higher mean score on the PSQI than married workers, but the difference was not significant.^38^ Furthermore, a study of HCWs in Turkey during the COVID‐19 pandemic found no significant relationship between poor sleep quality and marital status.^39^ The discrepancy in findings may be explained by work‐family conflict (WFC), which has been found to be higher among married HCWs (nurses and paramedical staff).^40^ WFC has been positively associated with emotional exhaustion,^41^ which in turn has been linked to sleep disturbances.^42^ Therefore, it is possible that the higher prevalence of poor sleep quality among married HCWs in the present study was due to the increased WFC and emotional exhaustion that often accompanies marriage and family responsibilities, particularly in healthcare settings where the demands of the job are high. Future studies could examine the impact of both marital quality and WFC on the quality of sleep among HCWs.

The present study found that the prevalence of poor sleep quality was highest among individuals with six to 12 children (84.6%), followed by those with one to five children (65.1%), and those with no children (51.1%). These findings highlight the significant impact of parenthood on sleep quality. A related study involving nurses in the US found that poorer‐than‐usual sleep quality was linked to experiencing more severe stressors the next day for parents, irrespective of whether they had one child, or two or more children. However, this association was not observed among nonparents. The study also found that the sleep‐stressor association was stronger for those with two or more children compared to those with one child.^43^ Additionally, research conducted in the United States showed that on average, parents who raise children (from birth through to the age of 18 years) lose 645 h of sleep time over this period when compared to those with no children.^44^

The findings of the present study indicated that HCWs other than doctors were significantly more likely to experience poor sleep quality compared to doctors. The reason that the present study only categorized HCWs into two categories (doctors and “others”) was the low sample size and many subcategories within this category. The reason that nondoctor HCWs had poor sleep quality is maybe due to their daily duties and low pay.^45^ These results are consistent with a study conducted in Bahrain, which found that being a nonphysician HCW was a predictor of both poor sleep quality and high perceived stress levels.^38^ Similarly, a study in the US found a significant association between being a nonphysician HCW and the risk of moderate to severe insomnia.^46^ One possible explanation for this disparity could be the difference in pay scales between doctors and other HCWs, given that doctors are better paid than other HCWs.

In the present study, a significant association was found between working night shifts and poor sleep quality among HCWs. The results showed that individuals who worked night shifts were twice as likely to report poor sleep quality compared to those who did not. These findings are consistent with similar studies conducted in Malaysia and Saudi Arabia, which also reported higher rates of poor sleep quality among night‐shift workers.^14^, ^47^ Furthermore, a study conducted in China demonstrated that nurses working consecutive nights or having night shifts also experienced poor sleep quality. Additionally, the study found that participants who worked consecutive night shifts showed a gradual decline in their sleep quality.^48^ These results are in line with the findings of the present study and suggest that poor sleep quality is a common problem among HCWs working night shifts. This is also supported by other studies in Taiwan and Italy which found that poor sleep quality was associated with working night shifts.^8^, ^49^ Moreover, the findings concur with all previously published reviews and worldwide meta‐analyses showing that nurses and/or HCWs that work night shifts have poor sleep quality.^33^, ^50^, ^51^, ^52^

In the present study, economic status was found to be another important factor linked to sleep quality. The findings indicated that 84.2% of participants with low income reported poor sleep quality, followed by 60.2% of those with middle income and 43.7% of those with high income. This result is consistent with the findings of a prior study conducted in the United States^22^ as well as a meta‐analysis of observational studies showing that poverty and low socioeconomic levels negatively affect sleep quality.^23^

Previous studies in China have shown that a traumatic or stressful event can disrupt sleep quality.^16^, ^17^ In the present study, 70.4% of individuals who experienced a traumatic event during the past month reported poor sleep quality, compared to 50.0% of individuals who did not experience a traumatic event in the past month. In relation to cigarette smoking, the findings did not indicate any significant association between cigarette smoking and sleep quality. This is consistent with a study conducted among Chinese adults.^20^ However, the results are different to a study conducted in Korea reporting that individuals who smoked cigarettes had lower sleep quality compared to those who did not.^20^

Previous studies have shown that male participants generally experience better sleep quality compared to female participants.^20^, ^23^, ^25^ Additionally, a survey study conducted in the United States found that female participants had higher odds of insomnia.^46^ However, the present study did not find any significant association between sleep quality and gender. Similarly, there was no significant association between age group and sleep quality, although a previous study in Egypt found that nurses younger than 30 years old were more likely to experience poor sleep quality. It is possible that other confounding variables, such as work schedule, work‐family conflict, stress level, and job satisfaction may explain these differences. Future studies should consider a range of potential confounding factors that could influence sleep quality among HCWs.

### 4.1 Strengths, limitations, and future directions

The present study is the first to examine sleep quality among HCWs in Afghanistan, offering unique insights into the sleep quality of HCWs in the country. It should also be noted that HCWs in Afghanistan have to work 24‐h shifts without any sleep. Moreover, Afghanistan does not have a government that is currently recognized internationally. While cross‐sectional, the study provides an important snapshot of the current state of sleep quality among Afghan HCWs. The sample was also representative of the Herat province HCW workforce.

The present study has several limitations that should be considered when interpreting the findings. For example, the sample size of the study may be considered relatively small, given the complexity of the research topic. Additionally, the study's sample was limited to HCWs from the province of Herat, which may not be representative of all HCWs in Afghanistan. The use of self‐report data may also introduce response bias into the results. Furthermore, the study only identified doctors and did not identify other HCWs by their specific roles, which is important because their sleep quality may vary based on their different job responsibilities and work‐related stressors. Future research in Afghanistan should explore potential factors that may affect the sleep quality of HCWs including work‐family conflict, job stressors, job satisfaction, and other potential variables to gain a more comprehensive understanding of the factors that influence sleep quality among this population. Another limitation of the study was using a nonvalidated version of the PSQI to assess sleep quality among HCWs.

It is recommended that future studies should also examine other potential confounding variables (e.g., such as an individuals' mental health states [depression, anxiety, stress], medications currently taken, late‐night leisure activities [e.g., excessive videogame playing, binge‐watching television shows, etc.]) that may affect overall sleep quality. This would further enhance the depth of understanding regarding the factors influencing sleep quality among HCWs. Furthermore, future research may benefit from incorporating a more nuanced examination of the intricate interplay between job demands, salary, working hours, shiftwork patterns, and levels of work‐related stress, to comprehensively address the multifaceted nature of sleep quality among HCWs.

## 5 CONCLUSION

The present study is the first attempt to evaluate the sleep quality of HCWs in Afghanistan. The findings provide valuable insights into sociodemographic factors associated with poor sleep quality among Afghan HCWs, including marital status, number of children, field of work, working night shifts, economic status, and the experience of a traumatic event in the past month. The study recommends the need for Afghan health employers to create a healthy work environment that prioritizes the well‐being of their employees by limiting overtime hours, providing rest breaks during long shifts, and ensuring that HCWs have access to necessary resources for coping with work‐related stressors. These policies would promote the overall health and well‐being of the workforce and would likely lead to better patient care outcomes.

## AUTHOR CONTRIBUTIONS

**Ahmad Shoaib Haidarzada**: Conceptualization; investigation; writing—review and editing. **Ahmad Neyazi**: Conceptualization; writing—original draft; writing—review and editing; methodology; formal analysis; project administration; funding acquisition; validation; visualization; software; resources. **Bijaya K. Padhi**: Conceptualization; writing—original draft; writing—review and editing. **Nosaibah Razaqi**: Data curation; writing—original draft; supervision. **Habibah Afzali**: Data curation; writing—original draft. **Raz Mohammad Tabib**: Data curation; writing—original draft. **Mahsa Ahmadi**: Writing—original draft; data curation. **Mehrab Neyazi**: Writing—original draft; data curation. **Mark D. Griffiths**: Writing—review and editing; conceptualization; methodology.

## CONFLICT OF INTEREST STATEMENT

The authors declare no conflicts of interest.

## TRANSPARENCY STATEMENT

The lead author Ahmad Neyazi affirms that this manuscript is an honest, accurate, and transparent account of the study being reported; that no important aspects of the study have been omitted; and that any discrepancies from the study as planned (and, if relevant, registered) have been explained.

## Data Availability

The data that support the findings of this study are available from the corresponding author upon reasonable request. The authors confirm that the data supporting the findings of this study are available within the article and its supporting information materials.
